# The Preparation and Chemical Structure Analysis of Novel POSS-Based Porous Materials

**DOI:** 10.3390/ma12121954

**Published:** 2019-06-17

**Authors:** Xiaomei Yang, Guangzhong Yin, Zhiyong Li, Pengfei Wu, Xiaopei Jin, Qifang Li

**Affiliations:** 1 State Key Laboratory of Biobased Fiber Manufacturing Technology, China Textile Academy, Beijing 100025, China; xiaomei_yang@126.com (X.Y.); poly0707@msn.com (Z.L.); wupengfei@cta.com.cn (P.W.); ajinxiaopei@163.com (X.J.); 2 College of Materials Science and Engineering, Beijing University of Chemical Technology, Beijing 100029, China

**Keywords:** octaphenylsilsesquioxanes (OPS), heptaphenyltricycloheptasiloxane trihydroxy silanol (T_7_-POSS), porous materials, Friedel-Crafts reaction, POSS

## Abstract

In this work, we reported the preparation and chemical analysis of novel polyhedral oligomeric silsesquioxane (POSS)-based porous materials, which were prepared according to Friedel-Crafts chloromethylation by using aluminum chloride as the catalyst and dichloromethane as the solvent. Through controlling the treatment solvent (water or methanol) and kinds of POSS, several materials with different morphologies were conveniently obtained. The chemical structure of porous materials was systematically characterized by Fourier-transform infrared (FTIR) spectra, ^29^Si Nuclear Magnetic Resonance (NMR), ^13^C NMR, and X-ray photoelectron spectroscopy (XPS). The samples were further characterized by X-ray diffraction (XRD), scanning electron microscopy (SEM), and thermogravimetric analysis (TGA) to study their crystallinity, morphology, and thermal properties, respectively. The work systematically demonstrated the chemical structure of the porous materials. Moreover, the advantages and disadvantages of the preparation method and typical properties of the material were evaluated through a comparative analysis with other related research works.

## 1. Introduction

Porous materials have been extensively studied for their broad application as filters [1], anodes (for microbial fuel cells [2] and lithium ion batteries [3]), electrodes [4,5] for solar cells, electrochemical energy storage [6], ultraviolet light-emitting diodes [7], and for oil-water separation [8]. Traditionally, the porous materials were made from metals or metal oxide, such as ZrO_2_ [9], TiO_2_ [10], and NiO [11]. Generally, there were several traditional methods to prepare porous materials, for example, the emulsion templating method [12], contemplate method [13], sol-gel approach [14,15,16], and so forth. Recent examples of the successful synthesis of macroporous materials using highly concentrated emulsions have been described [17,18]. In addition, controlled phase separation, induced by polymers such as poly (acrylamide) [19] and poly (propylene glycol) [20], is also a widely used method to produce porous materials. Overall, these types of study have received a tremendous amount of attention in recent years due to their remarkable and wide variety of applications, as mentioned above.

POSS is widely used in polymeric nanocomposite modification, e.g., POSS/polystyrene nanocomposites [21]. POSS-based porous materials, a novel class of organic-inorganic hybrid materials, show a high thermal stability and offer easy post-synthesis functionalization, opening a promising research area to produce practically effective materials [22]. Nowadays, more and more POSS-based porous networks are being reported with high surface areas by linking functionalized POSS units through diverse reactions. For example, Zhang et al. reported POSS-based porous materials by a hydrosilation reaction using octavinylsilsesquioxane (OVS) as a precursor [23]. Liu et al. prepared hybrid networks by using OVS via a Heck reaction [24,25]. They further reported a series of hybrid nanoporous Polystyrene (PS)-POSS based materials via the Friedel-Crafts reaction [26]. Moreover, POSS porous materials viologen-linked porous cationic frameworks induced by the Zincke reaction for efficient CO_2_ capture and conversion [27].

The functional group on the surface of the absorbents is one of the key factors for improving the separation efficiency, and scientists have made great efforts to obtain novel adsorbents with special functional groups which contain a high quality. Therefore, porous adsorption resins modified by special functional groups via the Friedel-Crafts reaction have high potential for applications in the separation and purification of natural products with crude extracts [28]. At present, OVS and octaphenylsilsesquioxanes (OPS) have been widely studied. Alternatively, we will describe a well-reported route to the formation of macroporous materials by the Friedel-Crafts chloromethylation reaction based on two kinds of POSS. We have chosen the heptaphenyltricycloheptasiloxane Trihydroxy Silanol (T_7_-POSS) and OPS as raw materials and anhydrous aluminum chloride (AlCl_3_) as a catalyst. According to the literature, this is a very direct, efficient, and simple method. This has stimulated our interest in continuing to explore relevant issues. Lots of reported works have not yet given the reader a systematic and intuitive image, especially for the chemical structures. Therefore, this study intends to enrich the family of POSS-based porous materials and systematically analyze the chemical structures. Moreover, through a comparison with previous reports, we hope to comprehensively grasp the advantages and disadvantages of this method.

## 2. Experimental Part

### 2.1. Materials

Phenyltrimethoxysilane (97%, Alfa Aesar, Haverhill, MA, USA) was used without further treatment. Aluminum chloride (AlCl_3_), sodium hydrate (NaOH), methanol, and anhydrous dichloromethane (DCM) were used as received from Beijing Chemical Works (Beijing, China). T_7_-POSS was synthesized according to our recent work [29].

#### 2.1.1. The Synthesis of OPS

Phenyltrimethoxysilane (10 g), tetrahydrofuran (THF, 120 mL), distilled water (1.2 g), and NaOH (0.9 g) were placed in a three-necked flask equipped with a reflux condenser and a magnetic stirrer. The mixture was refluxed in an oil bath at 65 °C for 5 h and then cooled to 25 °C for an additional 15 h with stirring. Ethylic acid was dropped into the mixture and white gel was obtained. After 5 days, the precipitate was collected and washed with THF, acetone, and ethanol at least two times, respectively. The white crystals were then dried in a vacuum at 45 °C for 24 h to afford the products with a yield of 30%.

#### 2.1.2. The Preparation of the Porous Materials

POSS-based porous materials were prepared by the Friedel-Crafts chloromethylation reaction. Typically, OPS (1.5 g), AlCl_3_ (7.2 g), and 80 mL anhydrous DCM were mixed into the three-neck flask and stirred continuously for 20 h at 40 °C. After that, the active hydrogen source was added dropwise into the reaction system. We could obtain different samples via different post-processing steps, as shown in Table 1. The released HCl gas was absorbed by saturated NaOH solution. When the gas release finished, the system was filtrated, giving rise to yellow-brown powder. Each sample was washed three times with ethanol and dried in the vacuum at 80 °C. The same procedure was used to prepare the porous materials based on the T_7_-POSS. Furthermore, in order to analyze the chemical structure, especially the existence of the Al element, H_2_SO_4_ (pH = 1) was used to treat sample A. Typically, sample A was impregnated in the H_2_SO_4_ solution for 20 h.

### 2.2. Characterization

Fourier-transform infrared spectroscopy (FTIR) spectra for all samples were obtained using KBr pellets in a TENSOR 27 spectrometer (Bruker, Billerica, MA, USA). Solid-state NMR was detected on the AV300 (Bruker, USA) at room temperature. Cross-polarization, magic spinning (CPMAS) ^13^C NMR was obtained at 11 kHz and the corresponding ^29^Si NMR spectra at 5 KHz TGA. Thermogravimetric analysis (TGA) was performed by using a TG209 (NETZSCH, Selb, Germany) in N_2_ atmosphere. Samples (5 mg) were heated from 50 °C to 800 °C at a rate of 10 °C·min^−1^. Two parameters were determined: temperature calculated at 10% (TG 90%) of weight loss and temperature at the maximum degradation rate (T_max_). Scanning electron microscopy (SEM) was performed on a HITACHI S-4700 scanning electron microscope (Hitachi, Tokyo, Japan) equipped with a field emission gun, at 4–10 kV. X-ray diffraction (XRD) patterns were recorded on a Bruker diffractometer with Cu Kα (wavelength: 1.5406 Å) radiation (Bruker, Billerica, MA, USA). The surface chemical composition of the porous materials was determined by an ESCA Lab 250 X-ray photoelectron spectrometer (XPS, Thermo Electron Corporation, Waltham, MA, USA) with an Al Ka X-ray source (1486.6 eV) at a constant record ratio of 40. The core-level signals were obtained at a photoelectron takeoff angle of 45° (with respect to the sample surface). The X-ray source was run at a reduced power of 150 W. Nitrogen sorption isotherm measurements were performed on a Quantachrome QuadraWin surface area analyzer (Quantachrome, Boynton Beach, FL, USA). Before measurement, samples were degassed at 100 °C for 24 h. A sample of 100 mg and an ultra high purity (UHP)-grade nitrogen gas source were used in the nitrogen sorption measurements at 77.3 K. S_BET_ was determined over a P/P_0_ range of 0.01 to 0.20. The pore size was calculated from the adsorption branch of the isotherm using the Barrett-Joyner-Halenda (BJH) method [18]. Total pore volumes (V_total_) were derived from nitrogen sorption isotherms at a relative pressure of P/P_0_ = 0.995.

## 3. Results and Discussion

### 3.1. The Preparation and Morphology Control of the Porous Materials

**Figure 1:**
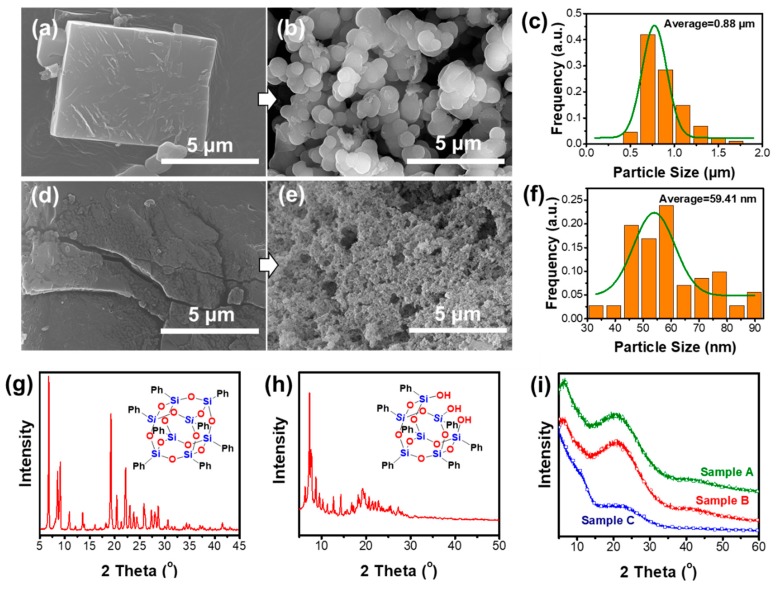
(**a**) Scanning electron microscopy (SEM) of original octaphenylsilsesquioxanes (OPS); (**b**) SEM of porous materials based on OPS, sample A; (**c**) particle size distribution of sample A; (**d**) SEM of original T_7_-POSS; (**e**) SEM of porous materials based on T_7_-POSS, sample C; (**f**) particle size distribution of sample C; and X-ray diffraction (XRD) curves of (**g**) OPS, (**h**) T_7_-POSS, and (**i**) the target samples. Particle size distribution is measured by Nano measurer 1.2 software based on SEM images [30].

The chemical characterization of the OPS is listed in the Supplementary Materials (Figure S1). The starting solutions were a yellow suspension (mainly due to the existence of AlCl_3_). As time elapsed, the mixture turned red and then became dark. Figure 1 shows an apparent difference in the surface morphology (See Figures S3 and S4 for more information on SEM morphology), particle size, and XRD diffraction due to the use of different kinds of POSS (T_7_-POSS and OPS). The smaller particles of sample C (particle size = 59.41 nm in average, Figure 1f) could have been caused by HCl gas evolution during the process because of the Si–OH. The HCl gas could have escaped from the T_7_-POSS-based materials, forming macro pores in the internal phase. However, OPS-based materials mainly release the HCl gas during the addition of deionized water or methanol after the Friedel-Crafts reaction, which gives rise to relatively large sphere-like particles (particle size = 0.88 μm in average, Figure 1c). Figure 1g,h show the XRD curves of OPS and T_7_-POSS, respectively. Both T_7_-POSS and OPS are white crystals. The crystallinity of OPS is better than that of T_7_-POSS because of its more symmetrical molecular structure. Moreover, we have found that all the obtained materials are insoluble in any solvents, which may due to the cross-linked internal structure. The insolubility of porous materials provided the possibility for their application in chromatographic separation as a filter. Herein, combined with the insolubility of the materials, it can be preliminary inferred that the interior of the materials is a random cross-linking structure, giving rise to an amorphous display, as seen in Figure 1i. Specific structures will be explained in the next section.

### 3.2. The Chemical Structure of the Porous Materials

#### 3.2.1. FTIR

**Figure 2:**
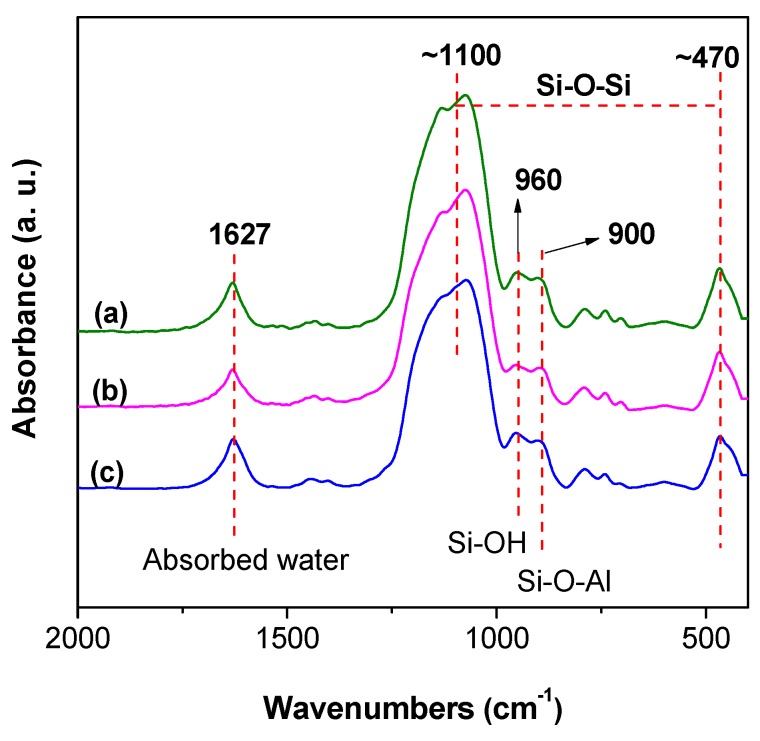
Fourier-transform infrared (FTIR) spectra of (**a**) sample A, (**b**) sample B, and (**c**) sample C.

As can be seen from Figure 2, the dominant Si–O–Si asymmetric stretching mode is split into doublets, which are located at about 1110 cm^−1^ and 1025 cm^−1^, and the band at ~500 cm^−1^ is exclusively due to an Si–O–Si symmetric stretching vibration [31]. There are several low-intensity bands resulting from the residual group of Si–OH. The Si–OH groups lead to a broad O–H stretching band ranging from 3100 cm^−1^ to 3600 cm^−1^ (Figure S2) and a small shoulder due to isolated Si–OH stretching at 960 cm^−1^. Additionally, the peaks at 960 cm^−1^ indicate that the hydrolysis partly happened during the reaction after adding the water or methanol. Moreover, in the spectra of all samples, a remarkable band, probably due to Si–O–Al vibrations [32], appears at about 900 cm^−1^, and with adsorbed water on the surface at 1627 cm^−1^, as also marked in Figure 2.

#### 3.2.2. NMR

**Figure 3:**
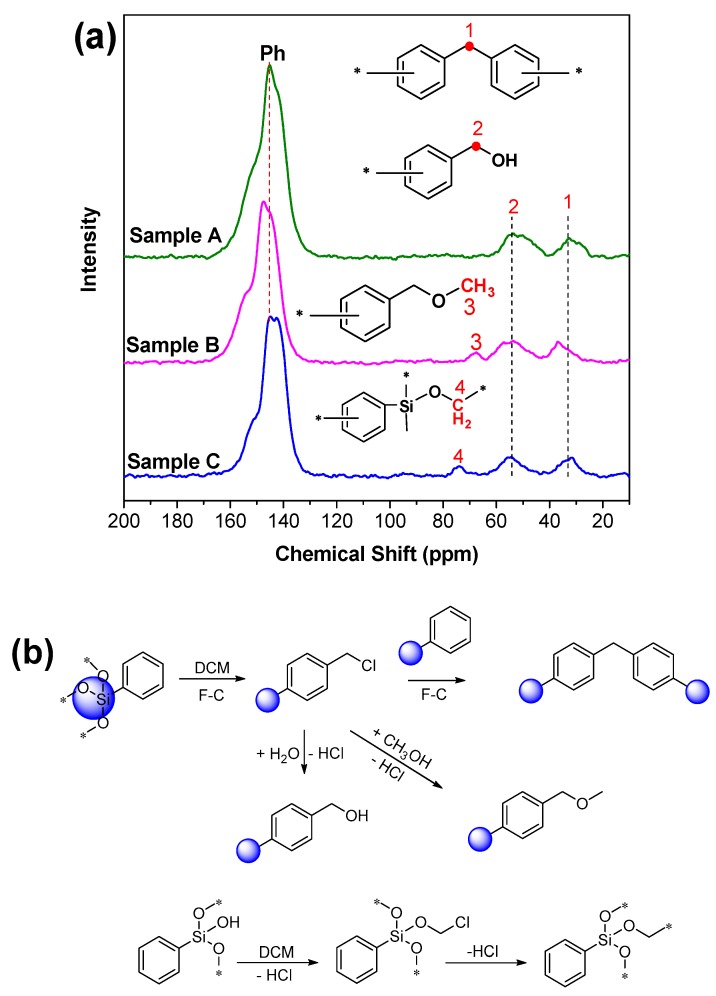
(**a**) Solid ^13^C NMR of sample A, sample B, and sample C, and (**b**) illustration of the typical structural formation. F-C means the Friedel-Crafts reaction.

Chemical changes brought about by the cross-linking process have been followed with solid ^13^C NMR spectroscopy. Figure 3 shows the ^13^C NMR spectra of the samples with peak assignments. We can observe the characteristic pattern of the Ph–C–Ph with a resonance at ~32 ppm (peak a). The terminal –OCH_3_ (peak c), Ph–CH_2_–OH (peak b), and Si–O–CH_2_– (peak d) were found at ~65 ppm, ~55 ppm, and ~75 ppm, respectively. For sample A and sample B, no obvious bubbles were formed at the initial stage of the reaction. A large amount of hydrogen chloride gas was released only after water or methanol was added. Under this strong acidic condition, the POSS cage was partially destroyed and generated Si–OH accordingly. For sample C, Si–OH already existed at the beginning, and the reaction involving active hydrogen persisted throughout the whole reaction process. Therefore, gases were generated throughout the whole reaction process. Combining the chemical structure of ^13^C NMR spectrum analysis with the mechanism of the Friedel-Crafts reaction, we speculate that there are elementary reactions (Figure 3b). Typically, the reaction process is a random combination of its elementary reactions, which occur simultaneously. For sample C, the whole preparation process is accompanied by hydrogen chloride gas spillover because of the active hydrogen in Si–OH, giving rise to a relatively vigorous reaction procedure and special structure (Si–O–C–*, peak d) accordingly.

**Figure 4:**
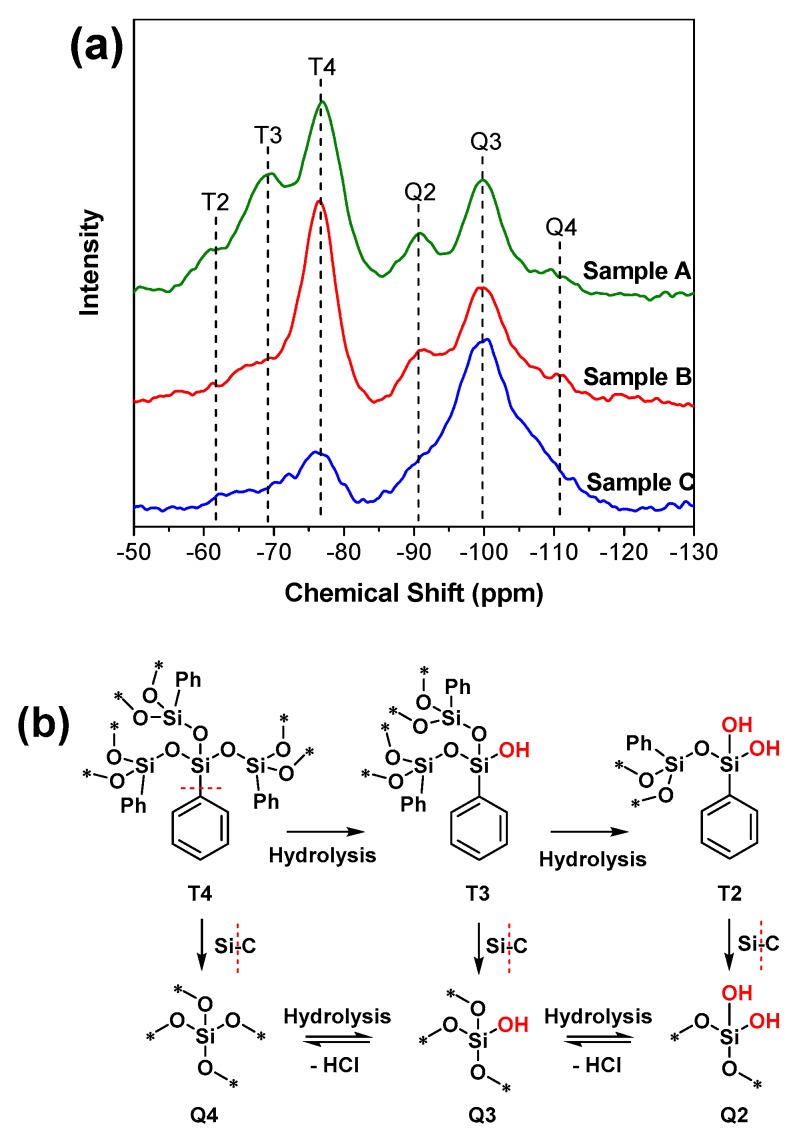
(**a**) Solid ^29^Si NMR of sample A, sample B, and sample C, and (**b**) the typical structures of the T-group and Q-group.

For ^29^Si NMR, relatively narrow chemical shift ranges for aryl (−70 ppm to −85 ppm) and siloxy (−95 ppm to −120 ppm) substituted POSS cages account for most of the data recorded [33]. In addition, the peak at ~−90 ppm is related to the presence of Q2 (Figure 4b). The peak at ~−100 ppm is assigned to silicon atoms bearing one hydroxyl group (called Q3). The peak at ~−110 ppm is due to silicon atoms without hydroxyl groups (called Q4) [34,35,36]. Accordingly, detailed analysis has been obtained, as shown in Figure 4a. Six peaks are recognized. The first group (T-group), at −62 ppm, −70 ppm, and −77 ppm, is attributed to the moiety of T2, T3, and T4 [37], respectively, which is in good agreement with the data mentioned earlier [35]. As for the second group (Q-group), the structure (Q2, Q3, and Q4) displays chemical shifts at −90 ppm, −99 ppm, and −112 ppm, respectively. Based on the ^29^Si NMR, we concluded that the structure of POSS was destroyed to some extent, namely, hydrolysis took place and provided an amount of terminal Si–OH on the surface of the materials, which agrees with the FTIR results. Furthermore, the existence of the Q-group indicates the cleavage of Si–Ph, ensuring silicon surrounded by four oxygen atoms, which is consistent with the report elsewhere [38]. The relative intensity of the Q-group was increased, indicating a higher hydrolysis existence in sample C. Overall, the NMR results confirm all the chemical circumstances of silicon. It is important to note that POSS has ideal structures to prepare porous materials, as mentioned, even though the cage structure was destroyed. That is to say, the hydrolysis of POSS will generate surface Si–OH, which provides special surface properties and further modification. The XRD spectra (Figure 1i) confirmed the amorphous structure of all samples. Herein, we can conclude that the inner structure of the materials is a mixture of the T-group and Q-group (Figure 4) formed via random chemical bonding. The typical chemical structures of the T- and Q-group are listed in Figure 4b. Notably, T4 can be transformed into T3, and the other evolutionary relationships between structures are also shown in Figure 4b. In brief, the T-group can transfer into the corresponding Q-group by breakage of the Si–C bond. A new T-group containing Si–OH can be obtained by hydrolysis of the Si–O–Si structure. Furthermore, Si–OH can be used as a source of active hydrogen to react with benzyl chloride in order to obtain the Si–O–C, which mainly exists in sample C. We further find that the ratio of T2 and T3 in sample A is much higher than that in sample B, indicating that there is a higher proportion of original POSS structure (T4) retention in sample B (comparing to sample A). In sample C, the Q-group peak is much higher than the T-group peak. Herein, we can see that the degree of destruction of the POSS by hydrolysis in the three materials follows the trend sample C > sample A > sample B.

#### 3.2.3. XPS

To obtain further insight, XPS was performed for identifying the elemental composition of the samples. The XPS C1s spectra were curve-resolved using peaks with an 80% Gaussian and 20% Lorentzian line shape and the full wave at half maximum (FWHM) of 1.91 eV, which was obtained from the analysis of the raw materials’ (T_7_-POSS and OPS) XPS spectra (Figure S5).

**Figure 5:**
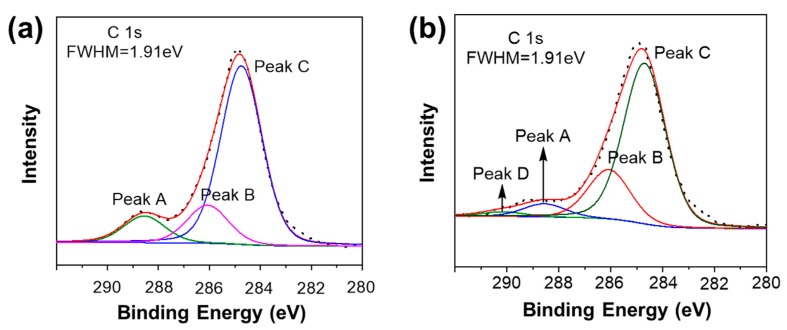
X-ray photoelectron spectroscopy (XPS) C1s of (**a**) sample A and (**b**) sample C.

Figure 5 shows the C1s core-level spectra. The C1s core-level spectra can be curve-fitted by three peak components with the binding energy (BE) at about 288.56, 286.07, and 284.66 eV, which is attributable to the Ph–CH_2_–OH, Ph–CH_2_–Ph, and C(Ph) species, respectively. In comparison with the C(Ph) species, the increase in BE of Ph–CH_2_–OH and Ph–CH_2_–Ph is attributed to the decrease of core electrons. The related data are summarized in Table 2. While the chloromethyl followed a careful washing procedure to remove the physical adsorbed regent with acid, distilled water, and ethanol until there was no white precipitate, an aqueous solution of silver nitrate was added to the filtrate [28]. As for sample C, a similar analysis of the XPS spectra can be seen in Figure 5b. Notably, the additional peak D in sample C is assigned to Si–OCH_2_–Ph, which is consistent with the ^13^C NMR results. The related data are also summarized in Table 2.

**Figure 6:**
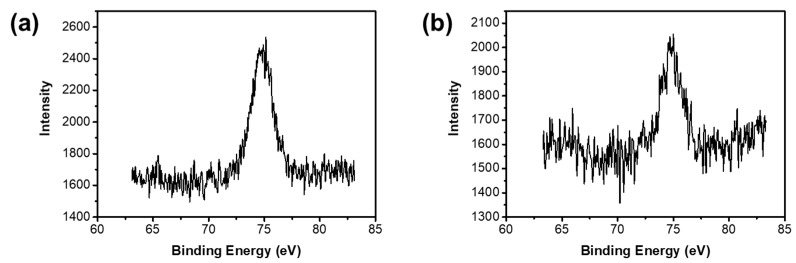
Al2p X-ray photoelectron spectroscopy (XPS) spectra of (**a**) sample A and (**b**) sample A with acid treatment.

The corresponding Al2p core-levels are also shown in Figure 6. The Al2p signal intensity of the samples without pickling is stronger, which may be due to the adsorption of a certain amount of Al on the surface. The Al on the surface of the materials can be partly removed via acid treatment. However, there is no remarkable content difference between the samples by acid treatment over 20 h, indicating that the Al residues are in the materials by chemical bonding (Si–O–Al), which agrees well with the FTIR results mentioned in Figure 2.

**Figure 7:**
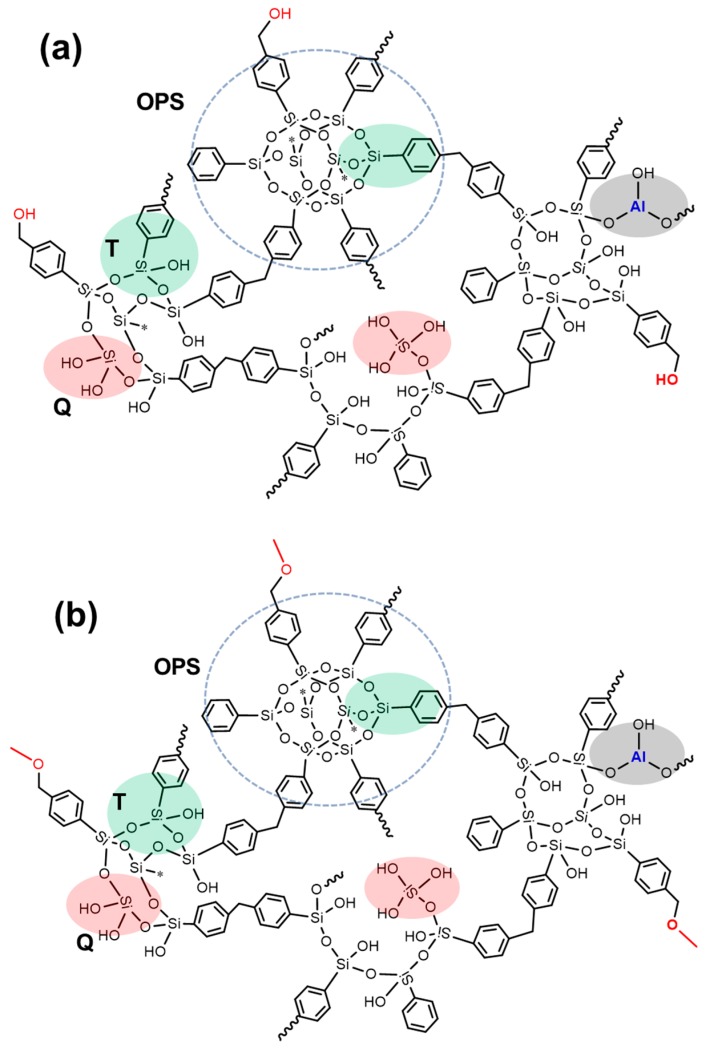

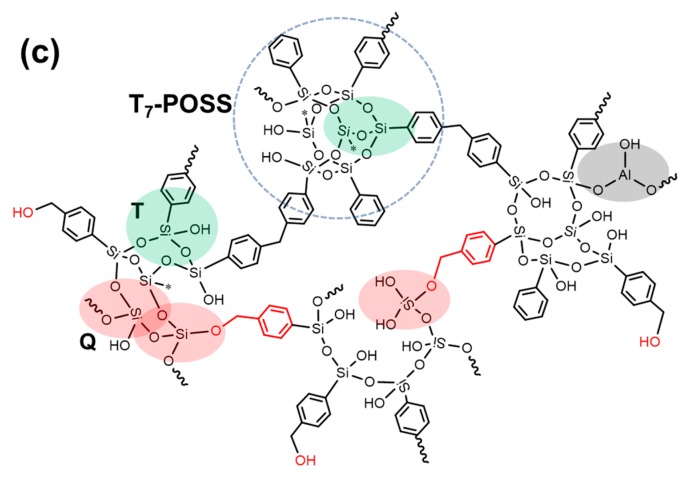
The expected chemical structure of the porous materials: (**a**) sample A, (**b**) sample B, and (**c**) sample C.

According to the XRD results, ^13^C NMR, ^29^Si NMR, and XPS analysis, we can see the specific chemical structures of the porous materials (as shown in Figure 7). In sample A, the T2, T3, T4, Q2, Q3, and Q4 coexist randomly with proper proportions. Because methanol provides active hydrogen, sample B contains a large amount of Si–O–CH_3_, resulting in a relatively low degree of hydrolysis (based on ^29^Si NMR spectroscopy). In addition to the above structure, a small number of Si–OCH_2_–Ph structures exist in sample C. Notably, T_7_-POSS-based porous materials formed the Q-group, which have higher contents than those of OPS-based porous materials.

### 3.3. Thermal Properties (TGA)

**Figure 8:**
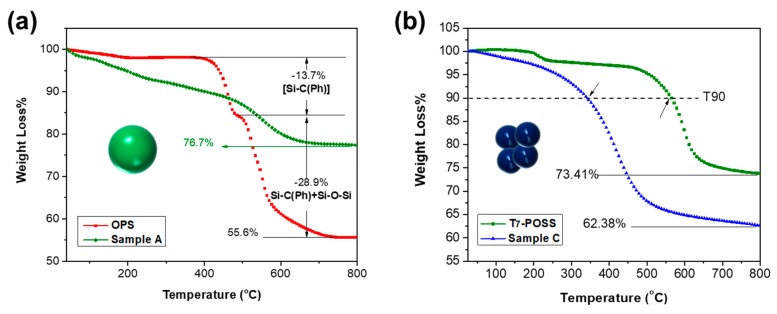
Thermogravimetric analysis (TGA) curves of (**a**) octaphenylsilsesquioxanes (OPS) and sample A, and (**b**) T_7_-POSS and sample C.

The thermal stability of POSS and its porous materials were recorded by TGA (Figure 8). The OPS showed an obvious loss near 450 °C, which was ascribed to the loss of the phenyl group. The OPS was reported to be stable by TGA and produced SiO_2_ at higher temperatures. The derivative thermogravimetry trace of OPS has been reported [33] to indicate rapid decomposition at 420 °C (T_95_), which agrees well with our result. Comparing sample C with sample A, the better thermal property of OPS-based materials compared to T_7_-POSS based materials is mainly attributed to the lager particle size, which leads to great differences in both heat and mass transfer. Moreover, it should be noted that the degradation at ~200 °C in T_7_-POSS is probably due to the intramolecular or intermolecular dehydration. The thermogravimetric curves decrease from the beginning (sample A and sample C), which is possibly due to the continuous release of adsorbed water on the materials’ surface (as evidenced by FTIR analysis). The typical data are listed in Table 3.

### 3.4. Further Discussion

We think that the HCl release is a typical drawback of the method. Due to the HCl release, a NaOH solution must be employed during synthesis to absorb the generated HCl. The HCl could cleave siloxane, which was also observed in previous POSS-based porous materials by some other reactions, e.g., Suzuki coupling [39], the Heck reaction [40], and so on. In other previous reports, OVS or OPS are usually used as raw materials. The obtained chemical structure and powder morphology are shown in Figure S6. Large-size random particles (except for Figure S6a) are obtained. Triethylamine and others can be added to the reaction system to absorb the hydrogen bromide gas generated by the reaction. We have found that the morphologies of materials in other reports are relatively random, while the materials prepared by OPS in this work form relatively regular spherical interfaces due to the rapid release of gases. For sample C, because the gas is continuously released during the whole reaction process, more surface forms, so the SEM shows a relatively high porous macrostructure.

We have listed the specific conditions required for the preparation of materials in a series of reports, including time, temperature, raw solvents, etc., in Table 4. From Table 4, we can see that the reaction temperature is relatively high in other previous works. In this work, the reaction could occur efficiently at a mild condition (namely, a low reaction temperature and AlCl_3_ being used as the only catalyst). CS_2_ (being toxic), DMF (with a high boiling point), tetrahydrofuran, and trichloromethane were usually selected as the solvent before. However, DCM chosen in this work can act as both a solvent and reagent, which is in line with the atomic economy. In addition, the use of T_7_-POSS raw material and the novel solvent choice finally extended the members of POSS-based porous materials.

Furthermore, we found that the specific surface area (S_BET_) of all the three samples is less than the value reported in the literature [24,25,26,37,39,40]. The specific curves and data of the porosity test are shown in Figure S7 and Table S1. Furthermore, we found that S_BET, sample B_ > S_BET, sample A_ > S_BET, sample C_, indicating that the higher the degree of hydrolysis POSS displays, the smaller the specific surface area. This may be attributed to the hydrolysis of POSS, which weakens the immobilization of rigid POSS molecules and leads to the collapse of internal voids. Therefore, OPS-based samples are higher than those of T_7_-POSS-based samples, and sample B is higher than that of sample A (because the hydrolysis degree of sample B is lower than that of sample A, as mentioned in the ^29^Si NMR section). In addition, sample C shows a remarkable porosity by SEM, but it has the smallest S_BET_. This indicates that the specific surface area of this kind of porous material mainly contributes to a mesoporous or microporous pore size. We can further infer that the S_BET_ values of the three samples in this work are lower than the values in other literature, mainly because of the high degree of POSS hydrolysis and the small space gap of only one –CH_2_– bridge. Finally, based on this work and the comparative analysis of existing literature, we can give some suggestions for the preparation and design of POSS-based porous materials, as follows: (1) maintain the integrity of POSS rigid particles as far as possible, which shows that adding acid binding agents such as triethylamine is an effective method; (2) reduce the use of volatile and toxic solvents such as CS_2_; and (3) introduce rigid bridging structures with a suitable spatial configuration.

## 4. Conclusions

We have demonstrated a facile synthetic method to prepare inexpensive porous materials by OPS and T_7_-POSS. The morphology and chemical structure of porous materials can be controlled by changing POSS types and post-treatment solvents. Through the analysis, we showed the chemical structure of the material, and pointed out the advantages and disadvantages of the material preparation method. Furthermore, the surfaces of the materials were shown to be reactive due to the Si-OH and can provide further functionalization. As produced, the materials described here could be used as adsorbents. Additionally, they possessed surface hydroxyl groups, which may attract much interest for practical applications, including catalysis [42]. Finally, based on this work and the comparative analysis of the existing literature, we can give some principles for the preparation and design of POSS-based porous materials, as follows: (1) maintain the integrity of POSS rigid particles as far as possible; (2) reduce the use of volatile and toxic solvents; and (3) introduce rigid bridging structures with a suitable spatial configuration.

## Figures and Tables

**Table 1:** The preparation of POSS-based porous materials.

Samples	Raw Materials	Reaction Time (h)	Active Hydrogen Source	Reaction Temperature (°C)
A	OPS	20	H_2_O	40
B	OPS	20	CH_3_OH	40
C	T_7_-POSS	20	H_2_O	40

**Table 2:** Corresponding C1s data of Figure 5.

Signal	Structure	Binding Energy (eV)
Peak A	Ph–CH_2_–OH	288.56
Peak B	Ph–CH_2_–Ph	286.07
Peak C	–C_6_H_6_	284.66
Peak D	Si–OCH_2_–Ph	290.15

**Table 3:** Corresponding data of Figure 8.

Samples	T_90_ (°C)	T_max_ (°C)
OPS	459.84	456.02/539.18
A	402.08	550.42
T_7_-POSS	564.50	211.45/597.73
C	343.47	420.91

**Table 4:** Reaction condition list.

No.	Reaction Time (h)	Reaction Temperature (°C)	Raw Materials (POSS)	Solvents	Other Conditions	References
1	48	80	OVS	THF/Et_3_N	Pd(OAc)_2_/PPh_3_	[24]
2	48	100	OVS	DMF/Et_3_N	Pd(OAc)_2_/P(o-CH_3_Ph)_3_	[25]
3	24	Reflux	OVS	CS_2_	AlCl_3_, Argon	[26]
4	48	100	OVS	DMF/Et_3_N	Pd(OAc)_2_/P(o-CH_3_Ph)_3_	[40]
5	24	60	OPS	CHCl_3_	AlCl_3_	[38]
6	24	Reflux	OVS	CS_2_	AlCl_3_, Stoichiometric benzene	[41]
7	20	40	OPS or T_7_-POSS	DCM	AlCl_3_	This work

## Supplementary Materials

The following are available online at https://www.mdpi.com/1996-1944/12/12/1954/s1, Figure S1: (a) FTIR spectrum and (b) ^29^Si NMR spectrum of Octaphenylsilsesquioxanes; Figure S2: Full FTIR spectra of (a) sample A, (b) sample B and (c) sample C; Figure S3: SEM morphology of T_7_-POSS based porous materials with different reaction time (a) 5 h and (b) 20 h; Figure S4: SEM morphology of OPS based porous materials with different reaction time (a) 5 h and (b) 20 h; Figure S5: XPS C1s of (A) OPS and (B) T7-POSS; Figure S6: Morphology and corresponding chemical structures. (a) is redrawn from reference [1] Copyright (2014) Royal Society of Chemistry, (b) is redrawn from reference [2] Copyright (2016) Royal Society of Chemistry, (c) is redrawn from reference [3] Copyright (2015) Royal Society of Chemistry, (d) is redrawn from reference [4] Copyright (2013) Royal Society of Chemistry, (e) is redrawn from reference [5] Copyright (2015) Royal Society of Chemistry, and (f) is redrawn from reference [6] Copyright (2014) Royal Society of Chemistry (The reference numbers are based on the Supplementary Materials ); Figure S7: Nitrogen adsorption and desorption isotherms for sample A, sample B and sample C; Table S1: Porosity data of sample A, sample B and sample C.
