# Supplementary material for: The Preparation and Chemical Structure Analysis of Novel POSS-Based Porous Materials
Source: Materials (Basel). 2019 Jun 17;12(12):1954. doi: 10.3390/ma12121954 (PMC6630320; doi:10.3390/ma12121954)
Supplement: Supplementary file 1 [file materials-12-01954-s001.pdf]

Supplementary Materials: The Preparation and Chemical Structure Analysis of Novel POSS-Based Porous Materials

Xiaomei Yang [1], Guangzhong Yin [1,*], Zhiyong Li [1], Pengfei Wu [1], Xiaopei Jin [1] and Qifang Li [2,*]

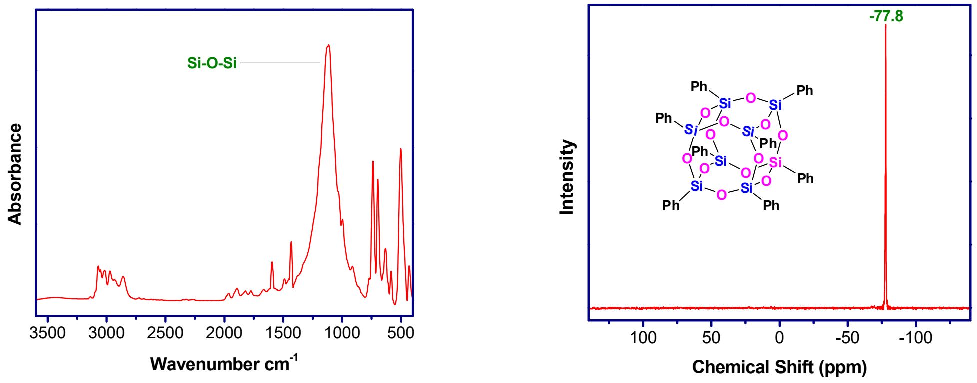

(**a**) (**b**)

Figure S1. (**a**) FTIR spectrum and (**b**) ^{29}Si NMR spectrum of Octaphenylsilsesquioxanes.

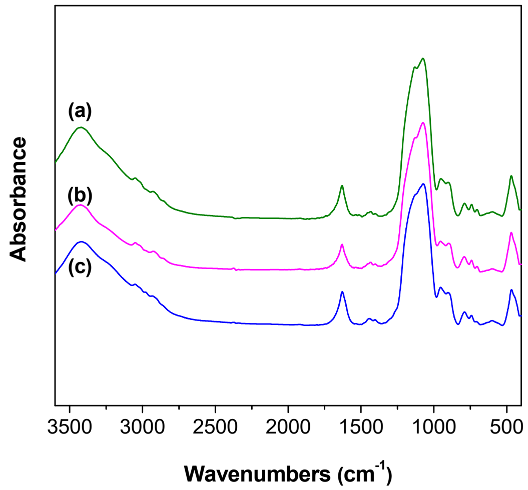

Figure S2. Full FTIR spectra of (**a**) sample A, (**b**) sample B and (**c**) sample C.

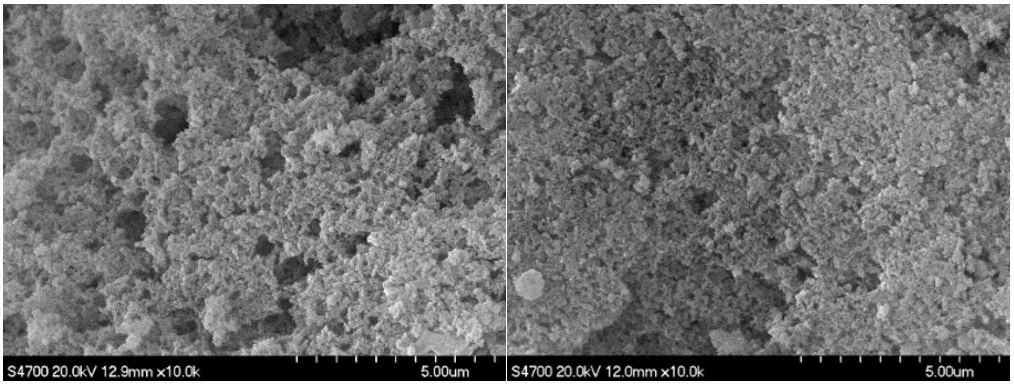

(**a**) (**b**)

Figure S3. SEM morphology of T7-POSS based porous materials with different reaction time (**a**) 5 h and (**b**) 20 h.

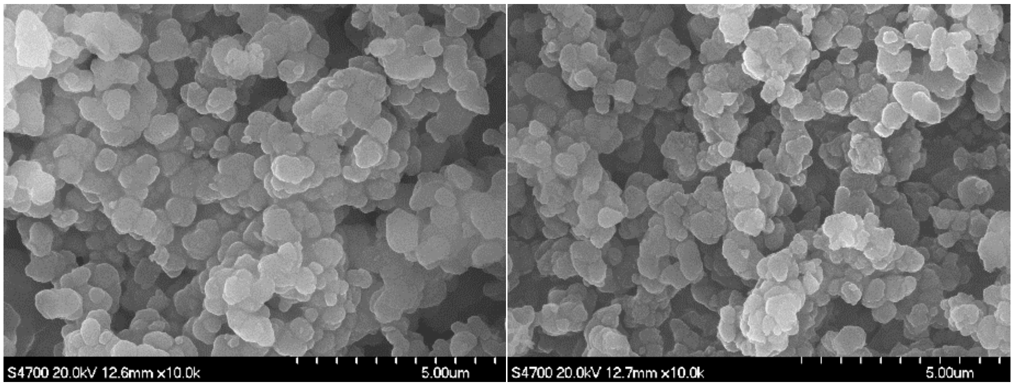

(**a**) (**b**)

Figure S4. SEM morphology of OPS based porous materials with different reaction time (**a**) 5 h and (**b**) 20 h.

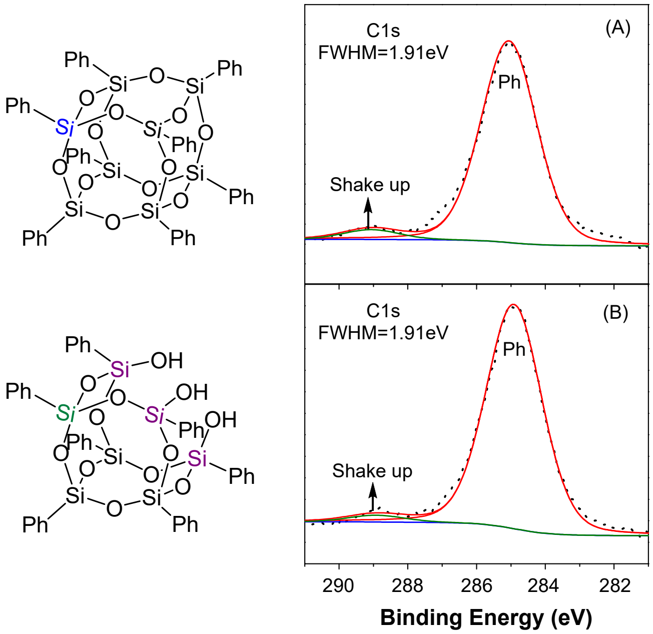

Figure S5. XPS C1s of (**A**) OPS and (**B**) T7-POSS.

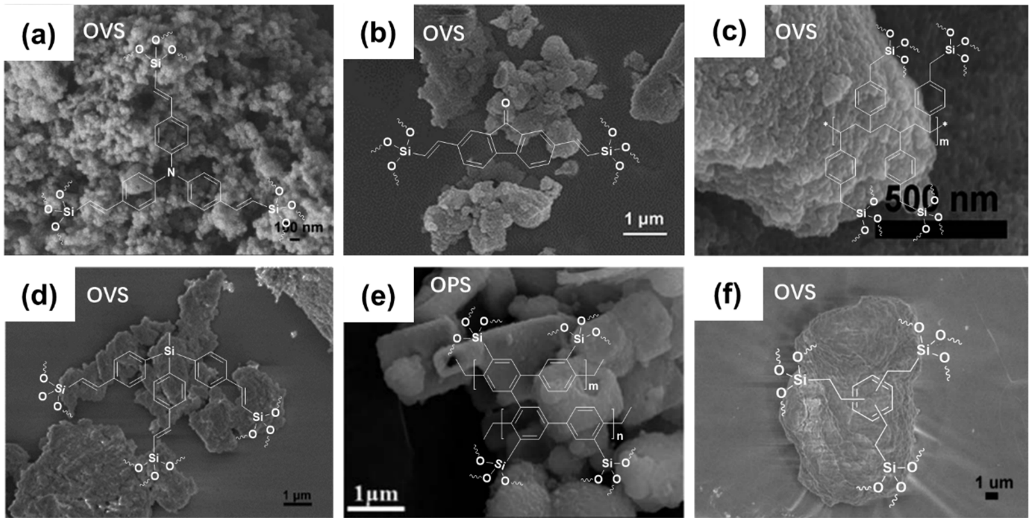

Figure S6. Morphology and corresponding chemical structures. (**a**) is redrawn from reference [1] Copyright (2014) Royal Society of Chemistry, (**b**) is redrawn from reference [2] Copyright (2016) Royal Society of Chemistry, (**c**) is redrawn from reference [3] Copyright (2015) Royal Society of Chemistry, (**d**) is redrawn from reference [4] Copyright (2013) Royal Society of Chemistry, (**e**) is redrawn from reference [5] Copyright (2015) Royal Society of Chemistry, and (**f**) is redrawn from reference [6] Copyright (2014) Royal Society of Chemistry.

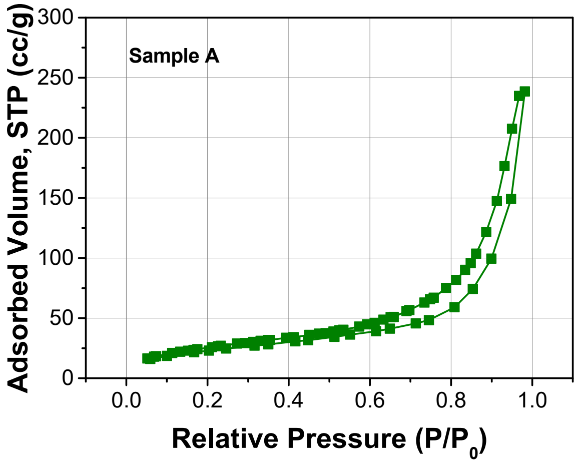

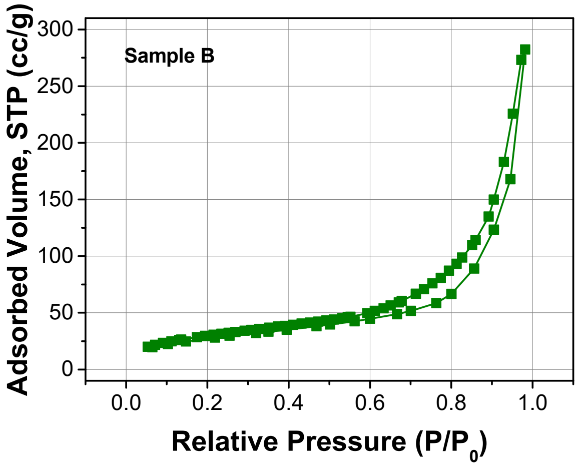

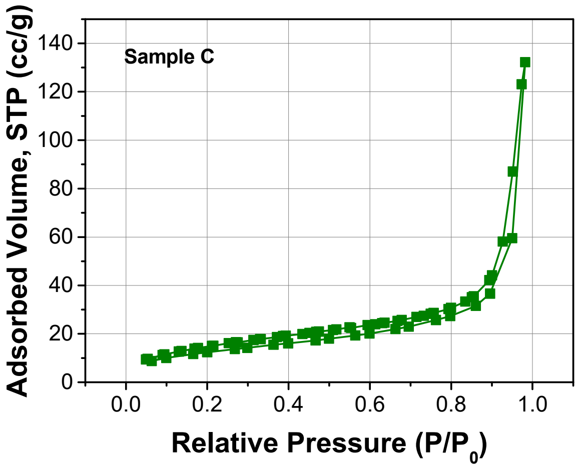

Figure S7. Nitrogen adsorption and desorption isotherms for sample A, sample B and sample C.

Table S1. Porosity data of sample A, sample B and sample C.

Sample No.	S_{BET} (m^2/g)	V_{total} (cc/g)	Pore Diameter (nm)
A	281.29	0.24	5.56
B	331.97	0.29	6.50
C	151.70	0.13	3.08

References

1. Wang, D.; Li, L.; Yang, W.; Zuo, Y.; Feng, S.; Liu, H. POSS-based luminescent porous polymers for carbon dioxide sorption and nitroaromatic explosives detection. *RSC Adv.* **2014**, *4*, 59877–59884.
2. Wang, D.; Yang, W.; Feng, S.; Liu, H. Amine post-functionalized POSS-based porous polymers exhibiting simultaneously enhanced porosity and carbon dioxide adsorption properties. *RSC Adv.* **2016**, *6*, 13749–13756.
3. Feng, S.; Wu, Y.; Li, L.; Yang, W.; Liu, H. Hybrid nanoporous polystyrene derived from cubic octavinylsilsesquioxane and commercial polystyrene via the Friedel–Crafts reaction. *RSC Adv.* **2015**, *5*, 12987–12993.
4. Wang, D.; Yang, W.; Li, L.; Zhao, X.; Feng, S.; Liu, H. Hybrid networks constructed from tetrahedral silicon-centered precursors and cubic POSS-based building blocks via Heck reaction: porosity, gas sorption, and luminescence. *J. Mater. Chem. A* **2013**, *1*, 13549.
5. Wang, S.; Tan, L.; Zhang, C.; Hussain, I. Novel POSS-based organic–inorganic hybrid porous materials by low cost strategies. *J. Mater. Chem. A* **2015**, *3*, 6542–6548.
6. Wu, Y.; Wang, D.; Li, L.; Yang, W.; Feng, S.; Liu, H. Hybrid porous polymers constructed from octavinylsilsesquioxane and benzene via Friedel–Crafts reaction: tunable porosity, gas sorption, and postfunctionalization. *J. Mater. Chem. A* **2014**, *2*, 2160–2167.
